# Longitudinal relationship between quality of life and negative life events among adolescents during COVID-19 pandemic: a cross-lagged panel analysis

**DOI:** 10.1265/ehpm.22-00284

**Published:** 2023-11-02

**Authors:** Yang Liu, Liya Deng, Ronghuinan Zhang, Yang Pu, Jie Yan, Hong Wang

**Affiliations:** School of Public Health, Research Center for Medicine and Social Development, Chongqing Medical University, Chongqing, China

**Keywords:** Quality of life, Negative life events, Cross-lagged panel analysis, COVID-19, Sex difference, Children and adolescents

## Abstract

**Background:**

The negative association of quality of life (QoL) and negative life evens (NLEs) among adolescents has been proved by cross-sectional studies, without exploring sex differences. This study aimed to explore the longitudinal associations between QoL and NLEs among adolescents during novel coronavirus disease 2019 (COVID-19) pandemic and whether there are sex or age differences.

**Methods:**

A stratified cluster sampling was used to select 1421 students in primary school and middle school in Chongqing, China. From November 2020 (T0) to December 2021 (T2), the Adolescent Self-Rating Life Events Checklist and the Adolescent Quality of Life Scale were used to collect 3 waves of data. The correlations between study variables were conducted by the Pearson correlation analyses. The direction and strength of the longitudinal associations were analyzed using cross-lagged panel analyses.

**Results:**

Results showed significant changes in both variables during COVID-19 pandemic (*P* < 0.001). Cross-sectional analyses showed stable negative correlations between NLEs and QoL stratified by sex or by age (*P* < 0.001). Sex and age differences in longitudinal relationships were shown by cross-lagged panel analyses. For males, NLEs had a short-term bi-directional association with QoL [β_A–D_ = −(0.091–0.340), *P* < 0.05]; for females, QoL had a short-term correlation with NLEs [β_A_ = −0.119), β_C_ = −0.109), *P* < 0.001]. In the youngest age group, NLEs had a short-term bi-directional correlation with QoL [β_A–D_ = −(0.098–0.428), *P* < 0.05]. There was a short-term association between total QoL and NLEs among students except the 14∼15 year group [β_A_ = −(0.071–0.149), β_C_ = −(0.086–0.119), *P* < 0.05], the long-term association between total QoL and NLEs was only significant in adolescents aged 14∼15 years (β_E_ = −0.132, *P* < 0.05). The strength of NLEs was slightly higher than that of total QoL, but lower than that of QoL in each dimension.

**Conclusion:**

There were negative longitudinal relationships between NLEs and QoL during COVID-19 pandemic, and the strength of the associations varied across sex or age. Strengthening QoL in different dimensions may be a promising way to reduce NLEs during the pandemic among adolescents, and interventions should be tailored according different sex and ages.

**Supplementary information:**

The online version contains supplementary material available at https://doi.org/10.1265/ehpm.22-00284.

## Background

In 1995, the World Health Organization (WHO) defines quality of life (QoL) criteria as an individual’s perception of their place in life in the context of the culture and value system in which they live and their goals, expectations, standards, and concerns [1]. It is generally considered to be a multidimensional structure that covers physical, mental and social functions and therefore represents the overall health of the individual [2], and its evaluation usually depends on individuals’ subjective evaluation of wellbeing and/or function in the different domains that make up the overall structure. Scholars found the importance of the assessment of QoL for children and adolescents year by year. Over the past few decades, QoL has gradually replaced traditional medical biological outcomes as the primary endpoint of medical and health research [3]. Study suggested that low levels of QoL are associated with poor physical and psychological developments, health status and lifestyle during childhood, adolescence and even adulthood [4]. Therefore, it is important to accurately assess the QoL of adolescents and identify the relevant variables to improve the health status of the young population.

On 11 March 2020, the World Health Organization characterized novel coronavirus disease 2019 (COVID-19) [5], a disease caused by the SARSCoV-2 virus, as a pandemic. Several public health precautions have been taken worldwide to slow infection rates, including isolation policies, extensive physical distancing, and closures of schools, resulting in a restructuring of everyday life [6]. On 13 August 2020 and 6 February 2021, the National Health Commission of the People’s Republic of China issued two versions of the “Technical Program for Prevention and Control of the COVID-19 in Primary and Secondary Schools in Autumn and Winter”, which specifies a number of control measures for primary and middle school students before and during the school year, such as relatively closed campus management, morning and afternoon checkups; regular health education and skills training, psychological support and guidance for students, etc [7, 8]. Since 24 January 2020, Chongqing took joint prevention and control measures, such as: wearing masks, suspending classes and work, not gathering crowds unnecessarily, closing extracurricular institutions, maintaining a safe distance of one meter, washing hands and disinfecting regularly, and paying attention to ventilation indoors, etc [9]. Since then, Chongqing has entered the stage of normalization of epidemic prevention and control, primary and middle school students gradually returned to study collectively. The prevalence of COVID-19 in Chongqing is at a low level, most of the time, the number of confirmed cases in the area is zero or single digits per day, according to news announced by the Chongqing municipal health and Health Committee [10]. However, it is important to note that adolescents have become one of the most vulnerable subgroups during the pandemic because of their immature immune system [11], lack of self-protection and weak psychological tolerance [12, 13]. Characteristics of COVID-19, such as acute onset, high mortality, long-term social isolation and its overwhelming negative news, also further aggravate impairment of QoL children [14]. A number of studies have confirmed the negative effects of COVID-19 on the QoL and mental health of adolescents [15–17]. More importantly, today, COVID-19 continues to threaten the QoL of teenagers [18].

Besides, Balkis and Duru supported that negative life events (NLEs) associated with COVID-19 and pandemic process experiences had a negative effect on the individual’s psychological health [19]. Until today, the impact of pandemic COVID-19 on NLEs is not yet understood sufficiently well, specifically in children and adolescents. Negative life events, such as interpersonal relationships, academic pressure, death of a relative, and having something valuable lost or stolen, are key risk factors for adolescent development and have a significant impact on the psychological and social outcomes of adolescents [20–24]. Compared with adults, adolescents’ perception of the environment is more sensitive, their self-assessment is also more vulnerable and easily to be influenced by the external factors [25]. It has been shown that the experience of NLEs in childhood can foster negative attitudes and biases about the self, and become activated by later adverse events impinging on the specific cognitive vulnerability, then lead to depression and other psychological problems in adolescence or adulthood [26]. Besides, higher rates of mental health problems were shown when multiple life events occurred together [27]. Moreover, according to contextual and behavioral psychotherapeutic approaches, the impact of life difficulties, failures or losses may be more determined by one’s ability to adaptively deal with those experiences rather than by the event itself [28, 29]. We can interpret this by the fact that some psychological processes may change the way people think about events that have occurred in their lives. Nolen-Hoeksema pointed out that women were better at seeking social support and more interpersonally oriented than men when faced with stressful events, moreover, they were more likely to report engaging in most types of emotion regulation [30]. Therefore, it is reasonable to suspect that there are likely sex differences in the occurrence of NLEs. Since it is not always possible to prevent NLEs in adolescents in a supportive way, it is, therefore, necessary to determine the variables that are related to NLEs among adolescents; this question also has important public health significance for facilitating students’ health during the epidemic quarantine.

Previous cross-sectional researches have examined the relationship between NLEs and QoL, although mediated by some variables, the occurrence of major NLEs directly predicted lower QoL. Cohrdes and Mauz suggested that a higher number of adverse childhood experiences (ACEs) were associated with poor mental health-related quality of life (HRQoL) [31]. There is a consistent body of evidence on the significant interference of events appraised as negative on individual quality of life, especially with psychological QoL [21, 32, 33]. To date, no studies, especially no prospective studies, were found that investigated the associations between the occurrence of negative life events and the impairment on QoL during the COVID-19 lockdown. In view of the limitations and research gaps in previous studies, we conducted a 1-year follow-up study to analyze the longitudinal relationship between NLEs and QoL during the COVID-19 pandemic among primary and junior middle students in Chongqing, China; and to explore the role of COVID-19 on NLEs and QoL among teenagers. Based on previous studies, we hypothesized that: (1) NLEs increase while QoL becomes lower during the past year; (2) NLEs are negatively correlated with QoL in cross-sectional analysis; (3) NLEs have bi-directional and longitudinal relationships with QoL; (4) the significance and strength of these longitudinal relationships are different in different sex and age groups, that is, there are sex and age differences of this relationship.

## Methods

### Sample size

The required sample size was calculated using the formula:

$$n={\left(\frac{Z_{\alpha /2}CV}{\varepsilon }\right)}^{2}$$

Previous studies showed that average quality of life score of adolescents in Chongqing was 141.19 ± 17.22 [34]. Therefore, in this study, CV = 17.22/141.19, α = 0.05, Z_α/2_ = 1.96, and ε = 0.01, resulting in a required sample size of 572. To control for invalid survey samples, we increased the sample size by 10%, resulting in 630 as the minimum sample size required for this study.

### Study design and participants

This is a 1-year longitudinal study of 1174 primary and junior high school students in Chongqing, China. The subjects of this study were adolescents in the period of puberty, and the age of puberty was about 10–19 years, considering the high course pressure and poor compliance of graduating students, only students in grades 4–5 in primary school and grades 7–8 in middle school were included in this study. The stratified cluster sampling method was used to select the study sample, two primary schools and two junior high schools were randomly selected from urban and rural areas of the region, respectively. The inclusion criterion was that students could understand and complete the questionnaire independently. The baseline survey was conducted in November 2020, with 1421 students completing an approximately 20-minute self-reported questionnaire in the classroom where they originally attended. A total of 1312 (92.33%) students completed the first follow-up visit in May 2021, and 1244 (87.54%) students completed the second follow-up visit in December 2021, the individuals lost to follow-up due to transfer, competition or illness at home. Finally, after excluding some questionnaires with poor quality or missing rates >10%, 1174 (82.62%) participants who provided data three times were included in the study.

### Procedure

The study was approved by the ethical committee of Chongqing Medical University in accordance with the Declaration of Helsinki and written informed consent was obtained from students and their parents before the research study began. One week before the survey, students were informed in advance by their teachers of the purpose of the survey and were assured that their responses would be confidential and anonymous. After students went home to seek consent from their parents or guardians, students signed an informed consent form on the day of the survey and completed the questionnaire voluntarily. We informed the participants about their right to withdraw from the survey at any time.

### Measurements

#### Negative life events

The Adolescent Self-Rating Life Events Checklist (ASLEC) was used to assess the impact of NLEs on adolescents over the past 3 months, taking into account the physiological and psychological characteristics and social role characteristics of Chinese adolescents [35, 36]. The scale has been widely used in the study of adolescent life events because of its specificity, simplicity and ease of use, and the validity and reliability of this instrument have been confirmed by several previous studies [14, 24, 36, 37]. The 27 items of the ASLEC are used to generate scores of five subscales: interpersonal relationship, study pressure, punishment, bereavement and health and adaptation. Previous studies have confirmed that each dimension of the scale has good reliability and feasibility and can be used as a stand-alone scale for surveys of adolescent populations [36]. Considering that the present study was exploring the longitudinal relationships between NLEs and QoL, bereavement events such as the death of relatives or friends are often unexpected and unpredictable, therefore, the loss dimension of ASLEC was excluded from the present study. This modified scale includes 20 items, such as: Have you failed or underachieved on a test during the past three months? Each item was assessed with a 5-point Likert scale, from 1 (no impact) to 5 (extremely heavy impact), according to the degree of physical and psychological impact on adolescents. A higher raw score represents a higher degree of the pressure and negative impact. In this study, the Cronbach’s alpha of this measure was 0.822 (95% *CI* [0.807, 0.836]).

#### Quality of life

The Adolescent Quality of Life Scale, consisted of 39 items, was divided into four subscales: physical (8 items), psychological (11 items), pubertal (6 items), and social (14 items) dimension (see Additional file 1), which were used to measure the QoL of children and adolescents in the past three months [38]. There were two types of items: the frequency of a certain phenomenon (type 1) and the satisfaction of participants with their life situation (type 2). Each item was rated on the 5-point Likert scale (type1: 5 = never, 1 = always; type2: 5 = very satisfied, 1 = very dissatisfied), with the values of item 23 reversed (5 = always, 1 = never), the test scores range from a minimum of 39 points to maximum 195 points, a high score represents a better quality of life. The previous researches have demonstrated the great applicability of this scale to both primary and middle school students [24, 34, 39, 40]. In this study, the overall Cronbach’s alpha was 0.914 (95% *CI* [0.907, 0.921]). The Cronbach’s alpha coefficients of physical, psychological, social, and pubertal were 0.851 (95% *CI* [0.838, 0.863]), 0.801 (95% *CI* [0.784, 0.818]), 0.872 (95% *CI* [0.861, 0.883]), and 0.613 (95% *CI* [0.578, 0.646]), respectively.

Besides, the following information was collected using a self-administered questionnaire: age (numerical variable), gender (male/female), and grade (4/5/7/8).

### Statistical methods

All data was entered into the Epidata 3.1 database and analyzed with SPSS 24.0 and AMOS 26.0. Descriptive statistics were calculated to describe the characteristics for the whole sample and stratified by sex or age. Data was presented as n (%) for categorical variables and mean (standard deviation, SD) for numerical variables. The Kolmogorov-Smirnov normality test was used, numerical variables conforming a normal distribution with equal variances were tested using t test, otherwise non-parametric test was used. Pearson correlation coefficients was used to test the association between NLEs and QoL. IBM SPSS AMOS 26.0 was used to perform structural equation modeling with maximum likelihood to test a cross-lagged panel model. The autoregressive coefficients of the same variable measured at different time points test the stability of the variables over time, and the cross-lagged coefficients of the pre-test and post-test of different variables test the longitudinal relationship between variables [41]. Such an association between variables, in which a variable x predicts future values of another variable y, is referred to as “Granger-causal” [42, 43]. Comparing the relative strength of the Granger-causal cross-lagged associations can provide direction for studying cross-lagged associations in more depth.

**Fig. 1:**
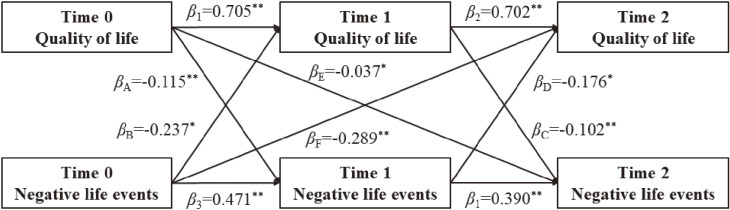
Cross-lagged panel model of NLEs and QoL.

In this study, we constructed 2 × 3 cross-lagged models to test the association of the two variables. Results were reported as standardized β coefficients. Due to observed sex differences regarding NLEs and QoL, analyses were stratified by sex (male/female) or age (∼10/∼11/∼12/∼13/∼14/∼15). As shown in Fig. 1, the model within this study captures the relationship between variables at baseline (in November 2020), time 1 (in May 2020) and time 2 (in December 2021): 1) Path A: association between QoL at baseline (QoL0) and NLEs at time 1 (NLEs1); 2) Path B: association between NLEs at baseline (NLEs0) and QoL at time 1 (QoL1); 3) Path C: association between QoL at time 1 (QoL1) and NLEs at time 2 (NLEs2); 4) Path D: association between NLEs at time 1 (NLEs1) and QoL at time 2 (QoL2); 5) Path E: association between QoL at baseline (QoL0) and the NLEs at time 2 (NLEs2); 6) Path F: association between NLEs at baseline (NLEs0) and QoL at time 2 (QoL2); 7) Paths 1–4: changes in quality of life or negative life events. Among them, the path A–D were considered as short-term relationships (6 months) and the path E–F were considered as long-term relationships (12 months). For the present study we were most interested in paths E and F, which test whether baseline NLEs were associated with changes in QoL after 1 year or vice versa.

Additionally, we conducted supplementary analyses to disentangle whether the relationships varied across sex or age, with exploring differences of QoL in each dimension. *P*-value (two-tailed) <0.05 was considered statistically significant.

## Results

### Descriptive data

**Fig. 2:**
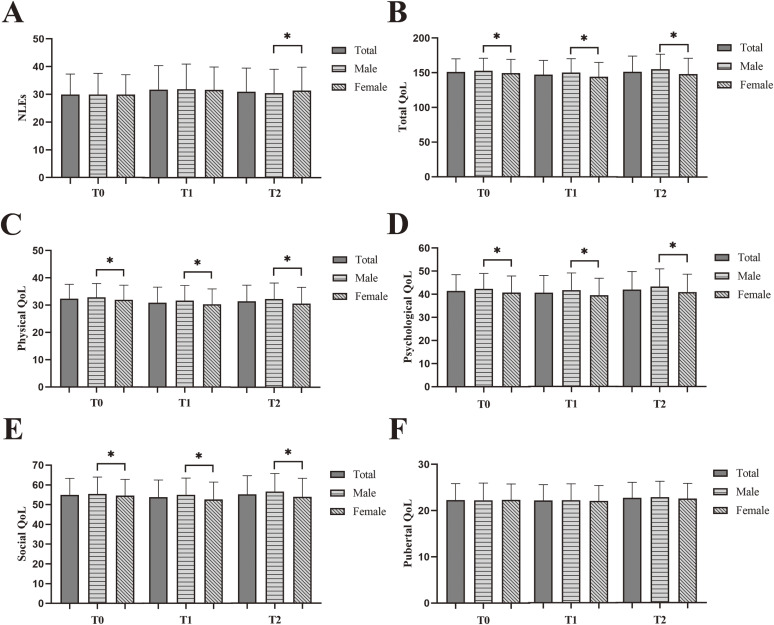
Comparison of NLEs and QoL in four different dimensions stratified by sex. A: negative life event; B: total physical quality of life; C: physical quality of life; D: psychological quality of life; E: social quality of life; F: pubertal quality of life. *: *P* < 0.05.

The sample consisted of 1174 participants, 562 (47.9%) were male and 612 (52.1%) were female, the average age was 12.02 ± 1.58 years at baseline, 12.49 ± 1.62 years at time 1, and 13.09 ± 1.61 years at time 2. The differences in NLEs, physical QoL, psychological QoL and social QoL scores by age group were statistically different except pubertal QoL score (Table 1). The score differences of NLEs and QoL between males and females were also compared, with statistically significant differences in all scores except pubertal QoL score (Fig. 2).

**Table 1:** Descriptive statistics of QoL and NLEs scores stratified by age.

	**Variable**	**Total ** **(n = 1174)**		**∼10 ** **(n = 145)**		**∼11 ** **(n = 281)**		**∼12 ** **(n = 101)**		**∼13 ** **(n = 261)**		**∼14 ** **(n = 333)**		**∼15 ** **(n = 98)**		**χ^2^**	***P***
						
		**Mean**	**SD**	**Mean**	**SD**	**Mean**	**SD**	**Mean**	**SD**	**Mean**	**SD**	**Mean**	**SD**	**Mean**	**SD**		
Time 0	Physical QoL	32.36	5.27	34.14	4.33	34.22	4.77	33.74	4.77	31.36	5.27	30.94	5.38	30.01	5.15	114.28	<0.001
	Psychological QoL	41.42	7.04	43.43	6.32	41.82	7.07	41.40	7.00	41.36	7.07	40.77	7.30	39.71	6.44	24.38	<0.001
	Social QoL	54.92	8.41	56.99	7.49	56.45	8.25	56.35	8.30	53.66	8.74	53.98	8.27	51.94	8.34	48.51	<0.001
	Pubertal QoL	22.26	3.56	21.61	3.76	21.94	3.82	22.49	3.70	22.31	3.33	22.64	3.35	22.54	3.42	8.17	0.147
	Total QoL	150.97	19.14	156.17	15.82	154.42	18.00	153.98	18.09	148.70	19.44	148.34	20.20	144.20	19.62	46.00	0.004
	NLEs	29.92	7.40	28.83	7.28	29.29	7.47	30.17	8.66	30.86	7.19	29.74	7.09	31.53	7.15	18.77	0.002
Time 1	Physical QoL	30.96	5.67	32.85	5.48	32.75	5.27	33.03	5.17	29.74	5.35	29.34	5.68	29.12	5.24	111.03	<0.001
	Psychological QoL	40.69	7.54	42.42	6.50	41.27	7.81	41.27	8.24	39.56	7.72	40.37	7.28	39.42	7.40	22.45	<0.001
	Social QoL	53.75	8.80	55.63	8.30	55.80	8.25	55.94	9.05	52.69	8.34	52.17	9.14	50.57	8.34	64.25	<0.001
	Pubertal QoL	22.08	3.43	21.27	3.58	21.50	3.60	23.01	3.09	22.43	3.27	22.32	3.41	22.36	2.98	26.35	<0.001
	Total QoL	147.48	20.84	152.17	18.22	151.32	20.26	153.25	21.12	144.42	20.76	144.21	21.22	141.46	20.33	52.87	<0.001
	NLEs	31.79	8.63	30.03	7.36	31.02	8.45	31.00	9.42	32.68	8.78	32.48	8.93	33.16	8.16	18.44	0.002
Time 2	Physical QoL	31.40	5.95	34.30	5.16	33.60	5.37	33.61	5.22	29.58	6.02	29.33	5.63	29.55	5.56	167.54	<0.001
	Psychological QoL	42.00	7.82	43.62	7.23	42.84	7.89	43.46	8.14	40.87	8.45	41.14	7.33	41.09	7.59	25.14	<0.001
	Social QoL	55.28	9.40	58.18	7.46	57.91	8.43	58.13	9.94	54.02	9.49	52.76	9.59	51.84	9.31	87.17	<0.001
	Pubertal QoL	22.67	3.36	22.55	3.22	22.77	3.37	22.93	3.06	22.84	3.46	22.54	3.40	22.37	3.46	3.45	0.632
	Total QoL	151.35	22.45	158.65	19.31	157.12	20.74	158.13	22.41	147.31	23.37	145.77	22.10	144.85	22.10	82.53	<0.001
	NLEs	30.98	8.53	29.61	7.62	29.98	8.42	29.24	8.53	32.41	9.18	31.40	8.08	33.06	8.18	25.94	<0.001

### Correlations analyses

Cross-sectional associations between NLEs and QoL at three time points were shown in Table 2. As previously assumed, in both the baseline and two follow-up surveys, NLEs were negatively associated not only with total QoL, but also with QoL in each dimension. A moderate negative correlation between total QoL and NLEs was evident at baseline and at both follow-up surveys in both male [*r*: −(0.651–0.738), *P* < 0.01] and female [*r*: −(0.623–0.706), *P* < 0.01] groups. After dividing QoL into four dimensions, the results were similar to the total QoL. NLEs were moderately negatively correlated with physical QoL (male: [*r*: −(0.510–0.605), *P* < 0.01], female: [*r*: −(0.451–0.575), *P* < 0.01]), psychological QoL (male: [*r*: −(0.572–0.687), *P* < 0.01], female: [*r*: −(0.545–0.654), *P* < 0.01]) and social QoL (male: [*r*: −(0.539–0.649), *P* < 0.01], female: [*r*: −(0.497–0.592), *P* < 0.01]), while the negative correlation was weak in the pubertal QoL (male: [*r*: −(0.320–0.444), *P* < 0.01], female: [*r*: −(0.281–0.400), *P* < 0.01]). The results of correlation stratified by age were similar to the above results stratified by sex. The results of collinearity diagnosis showed that the tolerance ranged from 0.522 to 0.700, and the expansion factor of variance (VIF) ranged from 1.429 to 1.912, which suggested that there was no multicollinearity between negative events and quality of life. Therefore, the longitudinal associations stratified by sex or age between NLEs and QoL in different dimension were considered in the following cross-lagged panel analysis.

**Table 2:** Pearson’s correlation analyses between NLEs and QoL in different dimensions.

	**Variable**	**Total**	**Sex**		**Age**					

			**Male**	**Female**	**∼10**	**∼11**	**∼12**	**∼13**	**∼14**	**∼15**
Time 0	Total QoL	−0.651*	−0.651*	−0.623*	−0.585*	−0.649*	−0.623*	−0.671*	−0.672*	−0.694*
	Physical QoL	−0.510*	−0.510*	−0.451*	−0.476*	−0.461*	−0.475*	−0.549*	−0.557*	−0.588*
	Psychological QoL	−0.572*	−0.572*	−0.545*	−0.400*	−0.538*	−0.546*	−0.622*	−0.641*	−0.647*
	Social QoL	−0.539*	−0.539*	−0.491*	−0.459*	−0.537*	−0.507*	−0.517*	−0.560*	−0.439*
	Pubertal QoL	−0.340*	−0.340*	−0.297*	−0.327*	−0.331*	−0.267*	−0.372*	−0.377*	−0.710*
Time 1	Total QoL	−0.674*	−0.674*	−0.658*	−0.699*	−0.737*	−0.737*	−0.671*	−0.587*	−0.639*
	Physical QoL	−0.559*	−0.559*	−0.547*	−0.560*	−0.614*	−0.597*	−0.688*	−0.478*	−0.623*
	Psychological QoL	−0.591*	−0.591*	−0.570*	−0.548*	−0.631*	−0.653*	−0.603*	−0.509*	−0.648*
	Social QoL	−0.606*	−0.606*	−0.588*	−0.635*	−0.669*	−0.672*	−0.597*	−0.547*	−0.495*
	Pubertal QoL	−0.320*	−0.320*	−0.281*	−0.232*	−0.346*	−0.331*	−0.490*	−0.303*	−0.273*
Time 2	Total QoL	−0.738*	−0.738*	−0.706*	−0.730*	−0.744*	−0.767*	−0.699*	−0.704*	−0.785*
	Physical QoL	−0.605*	−0.605*	−0.575*	−0.588*	−0.591*	−0.584*	−0.743*	−0.616*	−0.634*
	Psychological QoL	−0.687*	−0.687*	−0.654*	−0.645*	−0.659*	−0.714*	−0.715*	−0.666*	−0.727*
	Social QoL	−0.649*	−0.649*	−0.592*	−0.662*	−0.671*	−0.697*	−0.636*	−0.596*	−0.710*
	Pubertal QoL	−0.444*	−0.444*	−0.400*	−0.459*	−0.420*	−0.456*	−0.457*	−0.441*	−0.494*

*P < 0.01

### Path analyses

#### Autoregressive results

The autoregressive coefficients of NLEs and total QoL from T0 to T1 and T1 to T2 were all statistically significant (*P* < 0.05) in the whole group, both different sex groups and different age groups (Table 3). In addition, after dividing QoL into four dimensions, the autoregressive coefficients for each dimension remained significantly different (*P* < 0.001) for both the male and female groups (Table 4).

**Table 3:** Cross-lagged panel analyses of NLEs and total QoL stratified by age.

**Path**	**Total**		**Sex**				**Age**											

			**Male**		**Female**		**∼10**		**∼11**		**∼12**		**∼13**		**∼14**		**∼15**	

	**β**	***SE***	**β**	***SE***	**β**	***SE***	**β**	***SE***	**β**	***SE***	**β**	***SE***	**β**	***SE***	**β**	***SE***	**β**	***SE***
A	−0.115**	0.014	−0.116**	0.022	−0.119**	0.018	−0.126*	0.042	−0.115**	0.029	−0.130*	0.053	−0.149**	0.031	−0.071*	0.027	−0.101*	0.050
B	−0.237*	0.077	−0.340*	0.108	−0.170	0.190	−0.428*	0.204	−0.507*	0.164	−0.341	0.238	−0.312	0.168	0.123	0.153	−0.234	0.265
C	−0.102**	0.013	−0.091**	0.019	−0.109**	0.017	−0.098*	0.032	−0.119**	0.024	−0.101*	0.037	−0.103*	0.036	−0.086**	0.024	−0.083	0.052
D	−0.176*	0.061	−0.319**	0.085	−0.080	0.087	−0.356*	0.158	−0.225	0.121	0.011	0.177	−0.298	0.159	−0.065	0.116	−0.333	0.212
E	−0.037*	0.015	−0.030	0.023	−0.037	0.020	−0.059	0.039	−0.018	0.030	−0.063	0.047	0.007	0.042	−0.034	0.027	−0.132*	0.056
F	−0.289**	0.077	−0.323*	0.108	−0.270*	0.108	−0.268	0.172	−0.224	0.147	−0.722**	0.206	−0.102	0.207	−0.349*	0.156	−0.255	0.272
1	0.705**	0.030	0.647**	0.046	0.730**	0.039	0.567**	0.094	0.561**	0.068	0.644**	0.114	0.746**	0.062	0.790**	0.054	0.715**	0.097
2	0.702**	0.024	0.564**	0.036	0.791**	0.033	0.645**	0.065	0.653**	0.047	0.665**	0.074	0.724**	0.064	0.668**	0.045	0.664**	0.089
3	0.471**	0.036	0.463**	0.053	0.471**	0.049	0.277*	0.091	0.495**	0.069	0.459**	0.110	0.499**	0.083	0.587**	0.076	0.372*	0.137
4	0.390**	0.026	0.367**	0.037	0.429**	0.036	0.428**	0.071	0.369**	0.054	0.373**	0.074	0.505**	0.068	0.353**	0.045	0.349**	0.096

Path A: association between QoL at baseline and NLEs at time 1; Path B: association between NLEs at baseline and the QoL at time 1; Path C: association between QoL at time 1 and NLEs at time 2; Path D: association between NLEs at time 1 and the QoL at time 2; Path E: association between QoL at baseline and NLEs at time 2; Path F: association between NLEs at baseline and QoL at time 2; Path 1: change of QoL from baseline to time 1; Path 2: change of QoL from time 1 to time 2; Path 3: change of NLEs from baseline to time 1; Path 4: change of NLEs from time 1 to time 2.

**: *P* < 0.001, *: *P* < 0.05.

**Table 4:** Cross-lagged panel analyses of NLEs and QoL in different dimensions stratified by sex.

**Path**	**Physical QoL**				**Psychological QoL**				**Social QoL**				**Pubertal QoL**			

	**male**		**female**		**male**		**female**		**male**		**female**		**male**		**female**	

	***B***	***SE***	***B***	***SE***	***B***	***SE***	***B***	***SE***	***B***	***SE***	***B***	***SE***	***B***	***SE***	***B***	***SE***
A	−0.280**	0.070	−0.233**	0.059	−0.204**	0.057	−0.312**	0.044	−0.173**	0.043	−0.188**	0.039	−0.104	0.091	−0.188*	0.083
B	−0.117**	0.027	−0.058*	0.028	−0.152**	0.039	−0.115*	0.041	−0.205**	0.045	−0.162**	0.043	−0.066**	0.019	−0.059*	0.018
C	−0.225**	0.066	−0.216**	0.060	−0.125*	0.049	−0.203**	0.043	−0.190**	0.041	−0.191**	0.040	−0.172	0.092	−0.229*	0.086
D	−0.091**	0.024	−0.040	0.025	−0.099*	0.033	−0.146**	0.034	−0.170**	0.039	−0.052	0.039	−0.046*	0.017	−0.068**	0.017
E	−0.152*	0.075	−0.164*	0.066	−0.151*	0.057	−0.094	0.049	0.019	0.055	−0.082	0.045	−0.068	0.090	−0.111	0.085
F	−0.056	0.030	−0.057*	0.030	−0.140**	0.041	−0.132*	0.040	−0.117*	0.049	−0.090	0.048	−0.050*	0.021	−0.055*	0.020
1	0.584**	0.041	0.666**	0.037	0.558**	0.045	0.572**	0.040	0.471**	0.040	0.685**	0.037	0.339**	0.039	0.384**	0.037
2	0.570**	0.036	0.695**	0.033	0.486**	0.037	0.571**	0.033	0.490**	0.038	0.731**	0.034	0.305**	0.038	0.411**	0.035
3	0.549**	0.046	0.599**	0.044	0.536**	0.050	0.507**	0.045	0.538**	0.048	0.571**	0.045	0.618**	0.044	0.665**	0.040
4	0.423**	0.035	0.542**	0.034	0.428**	0.036	0.515**	0.036	0.432**	0.036	0.506**	0.034	0.507**	0.034	0.629**	0.032

Path A: association between QoL at baseline and NLEs at time 1; Path B: association between NLEs at baseline and the QoL at time 1; Path C: association between QoL at time 1 and NLEs at time 2; Path D: association between NLEs at time 1 and the QoL at time 2; Path E: association between QoL at baseline and NLEs at time 2; Path F: association between NLEs at baseline and QoL at time 2; Path 1: change of QoL from baseline to time 1; Path 2: change of QoL from time 1 to time 2; Path 3: change of NLEs from baseline to time 1; Path 4: change of NLEs from time 1 to time 2.

**: *P* < 0.001, *: *P* < 0.05.

#### Cross-lagged regression results

For the total QoL, all paths were all statistically significant (*P* < 0.05) for whole group. NLEs have a bi-directional longitudinal association with total QoL, both short-term (baseline to time 1 or time 1 to time 2) and long-term (baseline to time 2). But the results for males were different from those for females. Specifically, in male group, NLEs had a short-term bi-directional association with QoL [β_A–D_ = −(0.091–0.340), *P* < 0.05]; in female group, QoL had a short-term association with NLEs [β_A_ = −0.119, β_C_ = −0.109, *P* < 0.001]; NLEs had a long-term association with QoL for both males and females [β_F_ = −(0.270–0.323), *P* < 0.05]. The results also differed across the six age groups. In the youngest age group, NLEs had a bi-directional longitudinal association with total QoL, but only in the short term [β_A–D_ = −(0.098–0.428), *P* < 0.05]. There was only a short-term association between total QoL and NLEs among adolescents aged 10–11 and 12–13 years [β_A_ = −(0.115–0.149), β_C_ = −(0.103–0.119) *P* < 0.05], but conversely, no significant pathway was observed. Among adolescents aged 11–12 and 13–14 years, total QoL had a short-term association with NLEs [β_A_ = −(0.071–0.130), β_C_ = −(0.086–0.101), *P* < 0.05], whereas NLEs were associated with total QoL in the long term [β_F_ = −(0.349–0.722), *P* < 0.001]. Besides, the long-term association between total QoL and NLEs was only significant in adolescents aged 14–15 years [β_E_ = −0.132, *P* < 0.001]. Details are shown in Table 3.

After dividing QoL into four dimensions, the longitudinal relationship between QoL and NLEs differed by sex across dimensions. In the male group, psychological QoL had a bidirectional association with NLEs, not only in the short term but also in the long term (six pathways were all significant); both physical and social QoL had only a short-term bidirectional association with NLEs; and pubertal QoL had only a unidirectional relationship with NLEs, in other words, NLEs were associated with both short-term and long-term pubertal QoL. In the female group, psychological and pubertal QoL had a short-term bidirectional association with NLEs, whereas in physical dimension, this bidirectional relationship was long-term; social QoL had only a short-term association on NLEs (Table 4).

## Discussion

The present study investigated cross-lagged association between quality of life (QoL) and negative life events (NLEs) during COVID-19 pandemic among primary and junior high school students in Chongqing, China. To our knowledge, this is the first study to determine the direction of the association. The results of our longitudinal study showed that there were bi-directional relationships between NLEs and QoL, and these relationships were significantly negative. More importantly, we found sex and age differences in the significance and strength of the association between QoL and NLEs. In short, NLEs were both short-term and long-term associated with male total QoL, while they had only long-term association with females, in turn, total QoL had only a short-term association with NLEs for both males and females. QoL had a short-term association with NLEs in all age groups except the 14–15 age group, but the long-term association with NLEs was only observed in the 14–15 age group; NLEs had a short-term association with QoL except in the youngest two age groups, but the long-term association was only observed in the 11–12 and 13–14 age groups. In addition, the significance and strength of correlation were not completely consistent between different dimensions of QoL and NLEs. The three waves of data showed significant changes in NLEs and QoL scores for adolescents during COVID-19 pandemic. Thus, our study provided additional and comprehensive evidence for the longitudinal relationship between NLEs and QoL. It is important to note, however, that a cross-lagged panel model was used to identify longitudinal and directional associations between NLEs and QoL, not causal relationships. Given that there are no longitudinal studies of them, results must be interpreted with caution.

The stronger strength of NLEs on total QoL in males than in females can be explained by social support theory, which states that social support may influence adolescents’ assessment of situations, improve their problem-solving skills, and promote adaptive behavior. Most negative life events require individuals to make extensive behavioral readjustments in their daily lives [24]. An overload of such changes during a short period of time, especially during COVID-19 pandemic, may severely burden the individual’s coping abilities [44]. A study demonstrated that social support was a mediator buffering the negative impact of NLEs on QoL in adolescents [24]. It is possible that compared with females, males are less inclined to seek social support and they are more likely to suppress or avoid emotional expression [30, 45]. During the COVID-19 pandemic, home isolation reduced opportunities for face-to-face interaction and increased time spent on electronic devices, and females preferred socializing and texting, while male teenagers were more prone to play games [46]. It is precisely because males are not good at seeking social support and less interpersonally oriented that their ability to resist negative life events is poor. The negative effects of NLEs, such as reducing QoL, are displayed in a short period of time.

After stratifying by age, short-term association between NLEs and QoL was observed in the youngest age group and long-term association between NLEs and QoL was observed in the other 2 older age groups. This suggests that for younger adolescents, the decrease in QoL caused by NLEs may show up in a short period of time. Schools and parents should focus on NLEs in early adolescence and take relevant interventions as early as possible. Other related studies have also shown differences in the scores and effects of NLEs in different age groups of adolescents, Mann et al. found that the mean score on the NLEs scales rose with ascending age for both males and females, and that younger adolescents were more vulnerable to the impact of recent NLEs, resulting poor health, including depression, anxiety and other adverse emotions, even when the intensity of these NLEs was relatively low [47]. These differences may be related to the physiological and psychological developmental trajectories of adolescents. Physiologically, as the prefrontal cortex develops, older adolescents seem better able to override their initial fight-or-flight reactions and initiate more sophisticated responses to NLEs [48]. Psychologically, younger adolescents have immature cognitive abilities, including emotion regulation and impulse control, insufficient accumulated life experience and concomitant perspectives, and therefore would have higher levels of emotional vulnerability such as posttraumatic stress symptoms and depression after experiencing negative events [49].

Pubescent children or adolescents are living in a status of developmental transition from children to adults, involving physical, psychological, and social upheavals [50]. Previous studies have indicated that the risk of anxiety, depression and susceptibility to poor QoL would increase with pubertal status in adolescents [51]. After stratifying by age, total QoL had a short-term effect on NLEs in almost all age groups of adolescents. The QoL is dynamically changing and influenced by external factors, which suggests that it is a good interventionable variable. Previous studies have proved that interventions for adolescents can effectively improve the QoL in several dimensions [34]. Peer attachment [52], psycho-emotional status [53], health self-perception [54], social skills [55], and health-related behaviors, such as physical activity [56], are strongly correlated with QoL in adolescents or children. Therefore, measures such as increasing social and family support, reducing negative psychological emotions, improving emotion management skills, learning health-related knowledge, and developing healthy habits among adolescents may be effective ways to improve the QoL of adolescents. In April 2020, the Ministry of Education issued “Guidance Suggestions for Strengthening Mental Health Education in Primary and Secondary Schools in the New School Year”, proposing to “strengthen home-school communication, guide family education and assist parents in building good parent-child relationships” [57]. In the context of the COVID-19 pandemic, the mental health service system in primary and middle schools has been adjusted accordingly, such as the development of online mental health group activities and online one-to-one counselling [57]. These measures may help young people maintain their physical and mental health and cope with stressful events to a certain extent.

After dividing QoL into four dimensions, NLEs had the strongest association with psychological QoL, followed by the physical QoL. A considerable body of the literature argued in favor of the finding that personal negative experiences such as injury and illness were associated with impaired psychological health, both on adult and adolescent [58, 59]. A study proposed that the occurrence of major life events appraised as negative directly predicted lower psychological QoL [33]. Additionally, studies showed that, negative life events were significantly associated with increased psychological distress, especially with health-related events such as self-suffered illness [22, 24, 60]. A study found that only particular NLEs had significantly detrimental impacts on physical health or psychological health [61]. This is consistent with our research results. Physiological quality of life and psychological quality of life are different dimensions, and the significance and intensity of their correlation with negative life events are not completely the same. The results of the social dimension showed that negative life events have long-term and short-term negative effects on the social quality of life in male group, but have no effect in female group, which was consistent with the previous discussion about social support, that is, males were better at seeking social support to reduce the negative impact of negative life events. As far as we know, previous studies pertaining to the QoL of adolescents did not explore pubertal dimensions [62], however, a randomized controlled trial showed interventions to improve the pubertal QoL of adolescents were feasible [34].

During the one year of the COVID-19 epidemic, there were significant changes in QoL and NLEs among adolescents, specifically, the highest NLEs scores and the lowest QoL scores were observed at the first follow-up, but QoL scores were still higher than those in previous surveys of junior high school students in Chongqing between 2016–2018, using the same scale [34, 39]. Several longitudinal studies showed that scores of HRQoL decreased during the COVID-19 lockdown [63, 64] because of lockdown-related changes in children’s and adolescent’s everyday life, including lack of in-person contact with classmates, friends, and teachers [65, 66]. Differently, a study found that QoL of children and adolescents remained at the same level as before the COVID-19 outbreak [67]. Moreover, Lu also found no deterioration in mental health status among Chinese new undergraduate students after three months of mass quarantine for COVID-19 [68]. Besides, two cross-sectional studies of Chinese adolescents during the COVID-19 pandemic reported mean ASLEC scores of 41.3–57.3 [14, 69], however, the NLEs scores in the present study cannot be compared with other studies because four dimensions of the ASLEC were used rather than the whole scale. Given the uncertain course of COVID-19 pandemic in months and years to come, such information is important for public health authorities, but also for educational institutions, which should consider responding to the NLEs of students during the COVID-19 pandemic and in relation to future uncertainty.

Although the reciprocal associations between NLEs and QoL, as well as the sex and age differences elucidated in the present study have valuable theoretical and practical implications, the findings should be interpreted in light of several limitations. Firstly, the 1-year follow-up period of the present study happened to include the COVID-19 pandemic, when we conducted the first survey, China had entered the stage of normalization of epidemic prevention and control, and none of the participants had ever been infected with COVID-19, however, the lack of a control group makes it difficult to determine whether it is the passage of time or the policy at the time of the survey that affected the results. Secondly, we employed traditional CLPM, which could have poor performance when there was correlated trait variance in the constructs under investigation [70]. Thus, random-intercepts CLPM (RI-CLPM) is recommended to partial out any between-person differences and ensure that the cross-lagged effects reliably reflect within person fluctuations [71]. Thirdly, since the main aim of the current study was to specifically explore the longitudinal association between NLEs and QoL, the parsimonious model examined in the current study was incomplete. The role of other potentially relevant variables that may contribute to explain the effect of negative life events on QoL should thus be explored further in future studies. Fourthly, this study did not correct for P value when using multiple hypothesis testing, thereby increasing the probability of making Type 1 errors, and it is recommended that future studies should make some corrections such as Bonferroni correction, False Discovery Rate correction. Finally, two model (RMSEA and CFI) yielded inconsistent qualitative descriptions, and we are not currently able to grapple with disagreement between the two fit indices or explain why the indices disagree and the implications of the disagreement. It is prudent to assume that the model fit is suboptimal and so the positive results need to be interpreted with caution.

## Conclusion

The cross-lagged panel model in the present study highlights that there are significant bi-directional relationships between quality of life (QoL) and negative life events (NLEs) during COVID-19 pandemic, although the strength and duration of the associations varies across sex, age or different dimensions of QoL. The results determine NLEs contribute slightly stronger to this relationship than total QoL, but lower than QoL in each dimension, and other potentially relevant variables should be explored further in future studies. Moreover, the study strongly suggests that strengthening QoL in different dimensions is a promising way to reduce the occurrence of NLEs, especially during the COVID-19 pandemic, and adolescents of different sex or age are at different risk of NLEs, theoretical and practical interventions should be tailored accordingly.

## Abbreviations

QoL: quality of life
NLEs: negative life evens
COVID-19: coronavirus disease 2019
WHO: World Health Organization
HRQoL: health-related quality of life
ASLEC: Adolescent Self-Rating Life Events Checklist
SD: standard deviation

## Supplementary information

**Additional file 1:** Quality of life Scale for children and adolescents.

## Declarations

### Availability of data and materials

The datasets analyzed during the current study are available from the corresponding author on reasonable request.

### Author details

School of Public Health, Research Center for Medicine and Social Development, Chongqing Medical University, No. 1, Yixueyuan Road, Chongqing 400016, China.
