# Supplementary material for: Longitudinal relationship between quality of life and negative life events among adolescents during COVID-19 pandemic: a cross-lagged panel analysis
Source: Environ Health Prev Med. 2023 Nov 2;28:67. doi: 10.1265/ehpm.22-00284 (PMC10636289; doi:10.1265/ehpm.22-00284)
Supplement: Supplementary file 1 — Additional file 1: Quality of life Scale for children and adolescents. [file ehpm-28-067-s001.pdf]

Quality of life Scale for children and adolescents

Please recall the frequency of the following situations and your feelings in the last three months, and tick the appropriate box according to your actual situation.

	Never	Almost	Sometimes	Often	Always
1.Did you have trouble falling asleep?					
2.Did you feel tired when you wake up in the morning?					
3.Did you have a poor mental state during the day and can't lift your spirits?					
4.Did you feel tightness in your chest?					
5. Did you feel stomach pain?					
6.Did you feel dizzy?					
7.Did you feel weak and powerless?					
8.Did you feel numbness or tingling in your hands and feet?					
9.Did you have no interest in anything?					
10.Did you have no confidence in yourself?					
11.Did you worry that you are not as good as other students?					
12.Did you regret what you have done?					
13.Did you worry that you can't do things well?					
14.Did you worry about whether others really like to play with you?					
15.Did you get nervous and anxious easily?					
16.Did you have an uncontrollable urge to hit someone?					
17.Did you feel that someone is laughing at you behind your back?					
18.Did you ever threaten					

people you know?					
19.Did you have a hard time controlling your temper?					
20.Did you never consider the feelings of others in doing things?					
21.Were you discriminated against or excluded by other students?					
22.Did you have difficulty getting along with your classmates?					
23.Did you actively want to learn about puberty?					
24.Did you get scared when you think about puberty?					
25.Does the thought of puberty bother you?					
	Very dissatisfied	Dissatisfied	Neither satisfied nor dissatisfied	Satisfied	Very satisfied
26.Were you satisfied with your appearance?					
27.Were you satisfied with your body type?					
28.Were you satisfied with your relationship with your parents?					
29.Were you satisfied with your position in the family?					
30.Were you satisfied with your living environment?					
31.Were you satisfied with your relationship with your classmates around you?					
32.Were you satisfied with the state of your classes?					
33.Were you satisfied with your motivation to participate in school activities?					
34.Were you satisfied with your initiative in studying?					
35.Were you satisfied with					

your school life?					
36.Were you satisfied with your ability to learn and accept new things?					
37.Were you satisfied with the knowledge you have acquired about puberty?					
38.Were you satisfied with the ways of acquiring knowledge about puberty?					
39.Were you satisfied with the way you get along with your classmates of the opposite sex?					
